# In Vitro Impact Testing to Simulate Implant-Supported Prosthesis Retrievability in Clinical Practice: Influence of Cement and Abutment Geometry

**DOI:** 10.3390/ma13071749

**Published:** 2020-04-09

**Authors:** Andrea T. Lugas, Mara Terzini, Elisabetta M. Zanetti, Gianmario Schierano, Carlo Manzella, Domenico Baldi, Cristina Bignardi, Alberto L. Audenino

**Affiliations:** 1 Department of Mechanical and Aerospace Engineering, Politecnico di Torino, 10129 Turin, Italy; 2 PolitoBIOMed Lab, Politecnico di Torino, 10129 Turin, Italy; 3 Department of Engineering, Università di Perugia, 06125 Perugia, Italy; 4 Department of Surgical Science, C.I.R. Dental School, Università di Torino, 10126 Turin, Italy; 5 Division of Prosthetic Dentistry, Department of Surgical Sciences (DISC), University of Genoa, 16132 Genoa, Italy

**Keywords:** dental implants, dental cements, abutments geometry, retrievability, Coronaflex

## Abstract

Cement-retained implant-supported prosthetics are gaining popularity compared to the alternative screw-retained type, a rise that serves to highlight the importance of retrievability. The aim of the present investigation is to determine the influence of luting agent, abutment height and taper angle on the retrievability of abutment–coping cementations. Abutments with different heights and tapers were screwed onto an implant and their cobalt-chrome copings were cemented on the abutments using three different luting agents. The removals were performed by means of Coronaflex^®^. The number of impulses and the forces were recorded and analyzed with a Kruskal–Wallis test. Harvard cement needed the highest number of impulses for retrieval, followed by Telio CS and Temp Bond. However, abutment height and taper showed a greater influence on the cap’s retrievability (*p* < 0.05). Long and tapered abutments provided the highest percentage of good retrievability. The influence of the luting agent and the abutment geometry on the cap’s retrieval performed by Coronaflex^®^ reflects data from literature about the influence of the same factor on the maximum force reached during uniaxial tensile tests. The impulse force was slightly affected by the same factors.

## 1. Introduction

Cement-retained implant-supported prostheses are well established treatments for partially edentulous patients. Many clinicians often prefer this treatment option over the alternative screw fixation prosthetic, for its superior characteristics in terms of ease of fabrication, esthetics, occlusion, incidence of loss of retention and passivity of fit [1,2,3,4]. Even though there is no evidence to date on whether one treatment option is better than the other, cement-retained restorations are gaining popularity [5,6]. The increasing employment of this procedure, however, has given rise to the problem of retrievability; when a cementation is performed, complications such as the abutment screw loosening, fracturing or infection of the implant site may occur [7], and an easy and safe crown retrieval may be required [8]. While this issue is not a main concern when permanently cementing fixed dental prostheses (FDPs) on natural teeth [9,10,11] or screwing crowns to implants, when a cementation is performed on implant abutments, the surrounding conditions and clinical implications change completely.

Several factors are involved in the procedure design in order to achieve a suitable compromise between stability of retention and retrievability [12]. It is perhaps too trivial to say that both restoration longevity and retrievability are strongly influenced by the luting agent [13,14,15,16,17]. Temporary cements (e.g., zinc oxide eugenol, ZOE) allow for crown removal without damaging the underlying implant, at the expense of poor mechanical properties, such as low tensile resistance or high solubility in the oral environment, and permanent cements (e.g., glass ionomer and zinc phosphate) are recommended for permanent restorations due to their high tensile resistance values [18]. Ideally, a cement should be retentive enough to keep the crown in place, but, in the meantime, weak enough to allow an easy removal if necessary [19]. Nevertheless, to date, it is challenging to state the optimal implant configuration, due to the many variables involved. In fact, shape and size of the transmucosal abutment also strongly affect the implant behavior [20]. The relationship that emerges from literature is an increase in the cement retention with decreasing taper and increasing height of the abutment [21,22,23,24,25,26].

A valid combination of implant parameters (luting agent, height and taper) can guarantee good resistance of the system under physiological conditions while permitting removal if necessary. However, at present, there are only fragmentary indications that do not fully cover the complexity that the clinician is faced with. Furthermore, most of the works reported in literature experimentally analyze the implant retention by uniaxial tensile tests [13,22,27,28,29], without focusing on the actual condition of the removal of the crown by means of removal devices. The present investigation aims to evaluate the influence of (1) the luting agent, (2) the abutment height and (3) the abutment taper angle on the retrievability of cement-retained implant-supported prosthetics. The tests were performed in clinic-like conditions, in a testing setup where it was possible to perform a realistic retrieval by applying impulsive stresses on the crown-mimicking copings [30].

## 2. Materials and Methods

### 2.1. Design of Experiments

The present investigation was conducted in accordance with design of experiments (DOE) guidelines [31]. DOE is the first-choice statistical technique used in cases when more than one factor influences a result because, handling multiple factors at the same time, it can highlight interactions that could be hidden when investigating one factor at a time. Indeed, it allows the planning of controlled tests aiming at the evaluation of the factors that control a dependent variable.

In the present study, a team of clinicians was defined in order to select the main factors influencing the implant retrievability, as will be detailed in the following section. Moreover, in order to provide a realistic analysis of implant retrievability, clinical aspects and standard procedures were specified, thus defining the test environment and the test protocol. In accordance with this choice, each step of the protocol was performed by professional clinicians with over 10 years of experience in a dental laboratory.

### 2.2. Study Variables

Three factors were considered for the study: the luting agent, the abutment height and the abutment taper angle. For each factor the extreme high and low levels were determined based on clinicians’ technical knowledge, and between these three levels were selected. In detail, three luting agents were used, ranging from provisional to definitive: zinc oxide-eugenol (Temp Bond, Kerr Italia, Salerno, Italy), a dual-curing eugenol-free luting composite (Telio CS Link, Ivoclar Vivadent, Leicester, England) and zinc-phosphate (Harvard Cement, Harvard Dental Company, Hoppegarten, Germany). Three levels were then selected for the abutment heights (5 mm, 7 mm and 9 mm), and the abutment taper angle (0°—equal to a cylindrical shape, 2° or 4°). Other variability factors, such as environment temperature and humidity during the cement mixing phase, were not directly measured, and their effect on the statistical analysis was limited through randomization and blocking [31]. However, cement mixing and application was performed in a conditioned dental laboratory, with no relevant temperature changes, thus ensuring clinic-like realistic cementations.

Two indexes (i.e., dependent variables) were devised in order to evaluate retrievability: the coping retrievability index, i.e., the percentage of copings removed within a determined range of impulse numbers, and the total force, i.e., the total amount of force delivered on the implant.

### 2.3. Testing Setup

**Figure 1:**
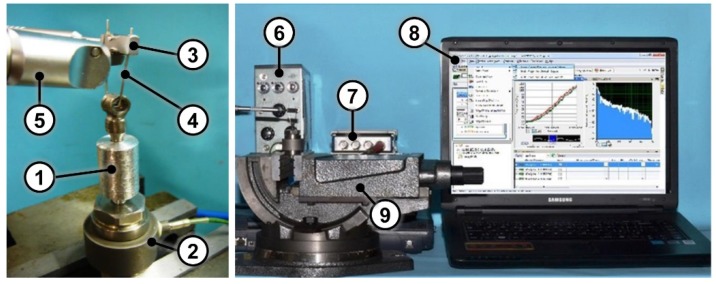
(**Left**) Close up view of the test setup with the cylindrical aluminum support (1), the load cell (2), the loop holder (3), the loop (4) and the Coronaflex^®^ (5); (**Right**) Test setup composed of the amplifier (6), the data acquisition board (7), the laptop equipped with LabVIEW SignalExpress (8) and the vise (9).

A custom manufactured cylindrical aluminum support (Ø = 14 mm, height = 20 mm) with a threaded housing for the implant was realized. It was screwed on a load cell (type 8210, Brüel & Kjær, Nærum, Denmark) for force measurement. An amplifier (type 2635, Brüel & Kjær, Nærum, Denmark) and a data acquisition board (NI 9234, National Instruments, Austin, Texas, USA) were used for the acquisition of measurement data with a 51.2 kHz acquisition frequency. The signals were visualized and acquired with LabVIEW SignalExpress (National Instruments, Austin, Texas, USA). The load cell was firmly secured in a vise before the tests (Figure 1).

### 2.4. Testing Protocol

**Figure 2:**
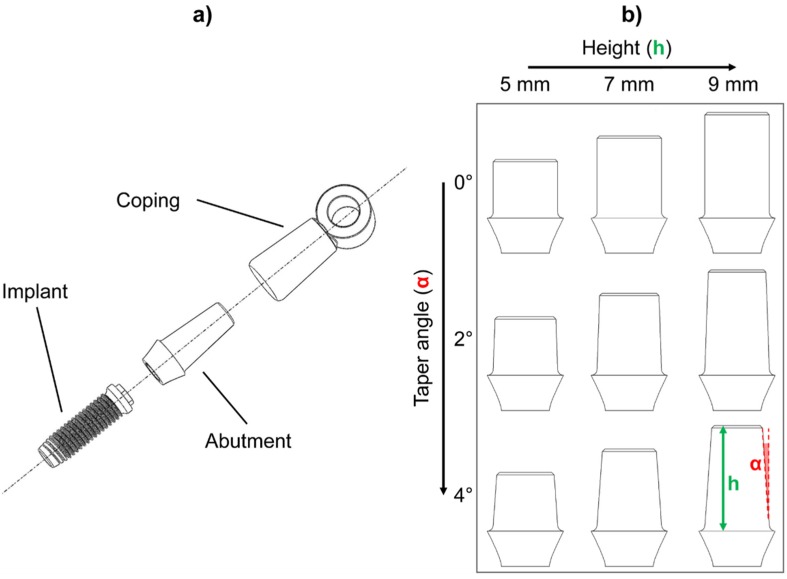
(**a**) Exploded view of the implant–abutment–coping assembly (9 mm–4° combination is shown as an example): the ring for the housing of the removal tool loop is positioned at the coping top. (**b**) The nine abutment geometries realized according to each height/taper angle combination. Height (h) variation is shown along rows while the taper angle (α) variation is shown along the columns.

Twenty-seven samples consisting of a titanium abutment and its cobalt-chrome coping were fabricated in accordance with the protocol described in a previous work [22]. Briefly, the abutments were designed with Procera^®^ CAD v.1.93 3D (Nobel Biocare AB, Procera^®^ by LT Elektroniklab. AB, Karlskoga, Sweden) and fabricated with a milling machine (Procera^®^, Nobel Biocare AB, Karlskoga, Sweden). A 0.1 mm die-spacer thermoplastic sheet and a 0.6 mm thermoplastic sheet for copings were adapted over each abutment. Waxing and casting were performed, and the abutment–coping fit was evaluated with silicon disclosing medium (Fit-Checker, GC Corp, Tokyo, Japan) under optical magnification (4X-300 Loupe, Carl Zeiss, Oberkochen, Germany). The upper part of each coping was equipped with a ring to house the removal tool loop. Figure 2a shows an exploded view of the implant–abutment–coping assembly for the 9 mm–4° combination. Three identical titanium abutments were produced for each combination of height and taper angle, thus obtaining three replicas for each of the nine height/taper angle combinations (9 = 3^2^: two factors, three levels) (Figure 2b). Each abutment was screwed onto an implant (Brånemark System Mk III TiUnite RP, diameter = 3.75 mm, length = 13 mm) and each group was luted with a different cement. All the cements were mixed and applied in accordance with the manufacturer’s instructions: Harvard Cement and Temp Bond were mixed by hand, while an auto-mixing double syringe was used for Telio CS Link. Cements were then painted onto the internal surface of the copings and applied on the abutments. All cement excess was removed, and the samples were left to rest for at least 3 days before the tests.

Coping extraction was performed by means of Coronaflex^®^ (KaVo Dental Excellence, Biberach/Riß, Germany). It is a device intended for extraction of crowns, bridges and temporary elements in dental medicine. When the operator triggers the impulse, compressed air pushes a piston towards a spring which activates the extractor tip. The impact lasts 8 µs, and the operating pressure can be selected in a range between 3 and 5 bar. The impulse force can be selected up to 4000 N using a specific control knob. Coronaflex^®^ is equipped with several accessories, including a loop and a loop holder, which were used in this study. In order to perform the removal, the loop was inserted in the coping ring and the Coronaflex^®^ tip was placed under the loop holder for impulse transmission (Figure 1). The tests were performed with the following working parameters: operating pressure = 4 bar; operating force = 4000 N.

Impulses were delivered by professional clinicians during all tests. The impulsive traction stress causes the fracture of the cement in the coping–abutment interface, allowing the retrieval of the coping. The number of impulses needed for complete removal as well as force trend over time were recorded.

After each test, the cement was removed from all parts with a dental spatula (ASA spatula 5100 Hylin, Asa Dental S.p.A., Lucca, Italy) and then with a cleaning solution (Jel-Sol, Dentaltorino, Torino, Italy) in an ultrasonic bath for 15 minutes. Copings were then rinsed and dried [22]. Each group was then cemented again with a different luting agent. A total of 224 tests were conducted.

### 2.5. Statistical Analysis

The tests were divided into 27 datasets based on the luting agent (Harvard, Telio CS and Temp Bond), the abutment height (5 mm, 7 mm and 9 mm) and taper angle (0°, 2° and 4°). The number of copings removed within a certain number of impulses and the sum of the impulsive loads applied during the retrieval were considered as retrievability indices for each dataset.

A Kolmogorov–Smirnov test was performed on each dataset using MATLAB 2019b (MathWorks, Inc., Natick, Massachusetts, USA) in order to determine whether the data were normally distributed. The null hypothesis of this test is that the data come from a standard normal distribution. Since it was not possible to reject the null hypothesis for any of the 27 datasets, three non-parametric Kruskal–Wallis (KW) tests were performed with MATLAB 2019b in order to assess whether (1) the luting agent, (2) the abutment height and (3) the taper angle had a statistically significative influence on the number of impulses and on the forces generated during coping removal. For this purpose, the data were divided into three groups for each KW test: for the first test, different cements were taken in consideration, obtaining a group for copings cemented with Harvard, one for Telio CS cementation and one for Temp Bond. In a similar way, the data were divided based on abutment height and taper angle for the two following KW tests. The test returns the *p*-value for the null hypotheses that the data in each group come from the same distribution. The significance level was set at 5%.

## 3. Results

**Figure 3:**
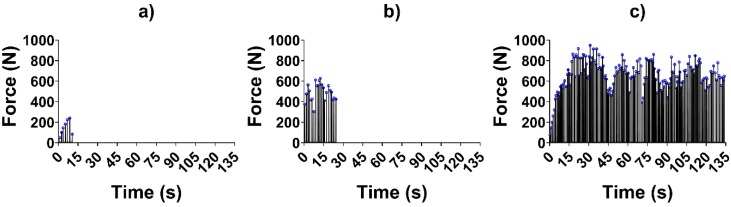
Force trends over time in three different tests: (**a**) Harvard cement, abutment height 5 mm, taper angle 4°; (**b**) Temp Bond, abutment height 9 mm, taper angle 2°; (**c**) Telio CS, abutment height 9 mm, taper angle 0°. Peak force values are highlighted in blue circles.

**Figure 4:**
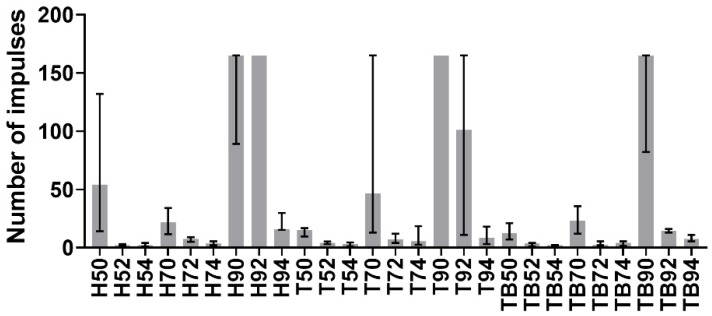
Medians of the number of impulses required for removal. The error bars represent the variability interval between the 25th and the 75th percentiles of each data group.

**Figure 5:**
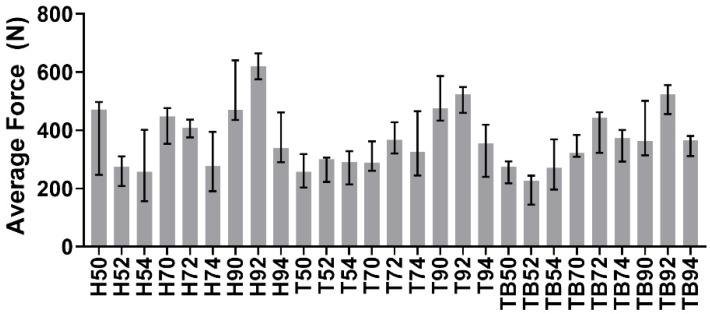
Medians and variability interval of average force value during the removals.

**Figure 6:**
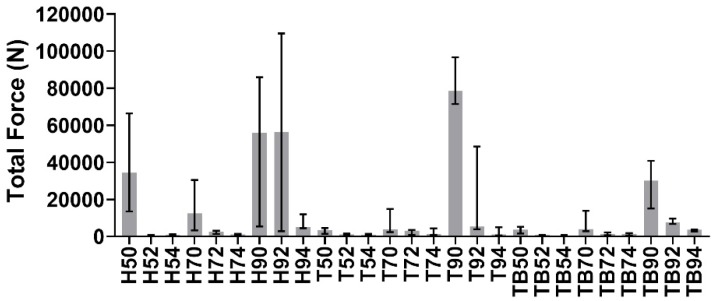
Medians and variability interval of the sum of peak forces measured during the removals.

Test signals were elaborated using MATLAB 2019b. The impulses delivered (i.e., the peaks in the force trend over time) were identified and counted for each signal, as shown in Figure 3. The average impulsive load was computed as the mean of the peak force values. The maximum number of impulses for the retrieval was set to 165; whenever a coping was not removed within 165 impulses, it was considered to be non-removable. As the tests were divided into 27 groups as described previously, in the following figures, the groups will be described by one (or two) letter(s), representing the luting agent (H, T or TB for Harvard, Telio CS and Temp Bond, respectively), and two integer numbers representing the abutment height (5 mm, 7 mm or 9 mm) and the taper angle (0°, 2° or 4°). As the Kolmogorov–Smirnov normality test highlighted the non-Gaussian distribution of the datasets, the median and field of variability of impulse number (Figure 4) and average force (Figure 5) were calculated for each group, rather than the means and standard deviations. Moreover, the sum of the peak force values measured during the tests was considered as the retrievability index. Median values and range of variability are shown in Figure 6.

**Figure 7:**
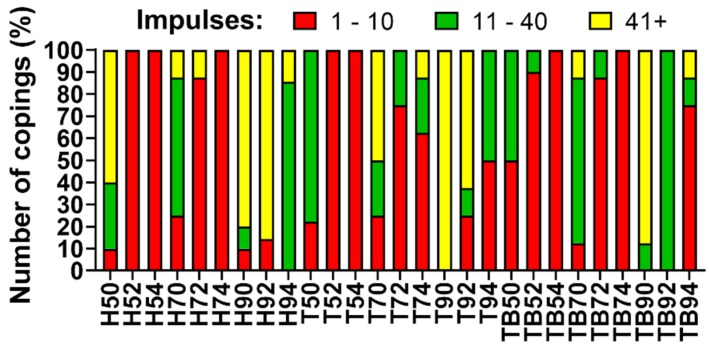
Copings retrievability index: observed percentage of instability (red), good retrievability (green) and no retrievability (yellow).

Because the optimal number of impulses for retrievability is around 30, retrievals requiring 11 to 40 impulses for the complete removal of the coping were considered optimal. The abutment–luting agent configurations requiring 10 or less impulses for removal were considered too weak, while copings removed with 41 to 165 impulses or not removed were considered to be non-removable, and therefore, not suitable for retrievable FDPs (Figure 7).

**Figure 8:**
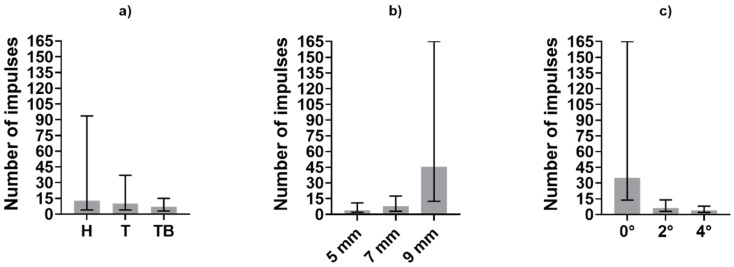
Medians and variability interval of impulses required for removal computed in subgroups divided according to (**a**) luting agent, (**b**) abutment height and (**c**) abutment taper angle.

**Figure 9:**
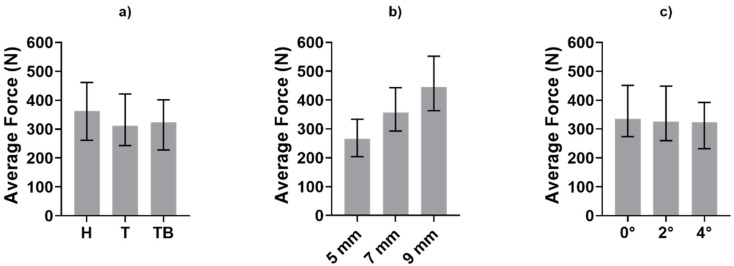
Medians and variability interval of average force value during the removals computed in subgroups divided according to (**a**) luting agent, (**b**) abutment height and (**c**) abutment taper angle.

Before the statistical analysis, the 224 tests were divided into three subgroups according to the three investigated factors (luting agent, abutment height and taper angle), as described in the previous section. Medians and variability range of the obtained dataset are shown in Figure 8 and Figure 9, in terms of impulse number and average force, respectively.

The influence of luting agents and abutment geometry on the number of impulses and average forces of the removals was evaluated by means of three Kruskal–Wallis tests; Table 1 and Table 2 show the *p*-values. Significative factors (*p* < 0.05) are underlined.

## 4. Discussion

Several previous studies described the influence of luting agents, abutment height and abutment taper on retention and retrievability of cemented FDPs. Uniaxial tensile tests were performed in most of those works, not fully representing realistic removal conditions. Indeed, retrievability should be evaluated with impulsive forces, which are more similar to the clinical approach [32]. The purpose of this study was to evaluate the influence of abutment geometry and luting agent on crown retention using Coronaflex^®^, which has already been proven to be efficient and reliable compared to alternative crown-removal tools in previous work [30,33].

At the same abutment height and luting agent, the number of impulses needed for the retrieval increases with the decrease of taper angle. This result, in accordance with literature data about uniaxial tensile tests, demonstrated that copings cemented on slightly tapered on non-tapered abutments are less retrievable. The only exception of this phenomenon can be noted comparing impulses number in luting agent-abutment geometry combinations referred to as H90 and H92 (Figure 4). However, it should be pointed out that the majority of Harvard-cemented copings on 9 mm long abutments with taper angles of 0° or 2° were not removed after 165 impulses, which was set as the retrievability limit. The dispersion of the H90 dataset could have been affected by a few badly cemented copings. The abutment height also affected the retrievability of the copings. An exception to the increase of the number of impulses with increasing height can be observed from Harvard-cemented copings on 5 and 7 mm abutments with a 0° taper. Again, the exception is observed together with highly dispersed data. However, both height and taper angle significantly influenced the number of impulses according to the KW test results, as shown in Table 1. In contrast with literature data, no significative differences were found between different luting agents (*p* > 0.05). Despite the lack of statistical significance, Figure 8 shows that Harvard-cemented copings required the highest number of impulses to be removed, followed by Telio CS- and Temp Bond-cemented ones, which is in accordance with the results of other studies in which the superior resistance of zinc phosphate cementations over zinc-oxide ones was established [13,15,16]. The influence of abutment height and taper also reflects previous findings [22,23,25]. In [22], a greater difference was detected between non-tapered and slightly tapered abutments than between abutments tapered with different angles. This phenomenon can also be observed in Figure 8, as the number of hits required in the case of a taper angle of 0° is considerably higher than in the other cases.

Figure 5 shows the medians and the range between the 25th and the 75th percentiles of the average force generated during each test. The load is not as heavily affected as the number of impulses by the luting agent and the abutment geometry. However, Figure 9 shows a monotonic trend for the abutment taper angle and height. This last factor influences the generated forces significatively (*p* < 0.05), as shown in Table 2.

**Figure 10:**
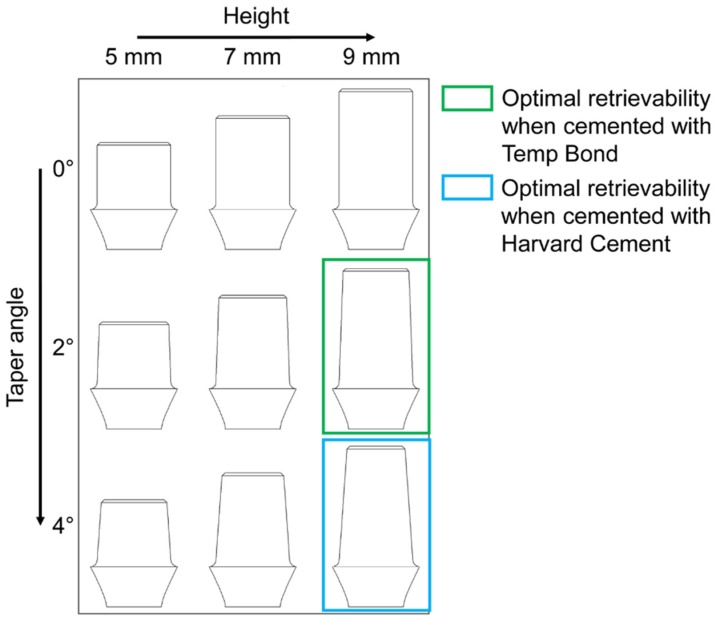
Optimal height–taper angle combinations resulted from the present study: the best percentages of good retrievability were obtained with Harvard-cemented copings on 9 mm and 4° abutments and with Temp Bond-cemented ones on 9 mm and 2° abutments.

Based on the number of impulses, the retrievability of the copings was classified as (1) instability, (2) good retrievability and (3) no retrievability (Figure 7). Testing performed on abutments shorter than 9 mm showed a non-negligible percentage of instability, in particular, when 5 and 7 mm height were combined with a tapered shape. In contrast, coupling abutments with a length of 9 mm and a taper angle of 0° provided non-removable copings. Therefore, the best combinations were found with a 9 mm length and taper-shaped abutments. In particular, the best percentages of good retrievability were obtained with Harvard-cemented copings on 9 mm and 4° abutments and with Temp Bond-cemented ones on 9 mm and 2° abutments (Figure 10).

The lack of retrievability highlighted in Figure 7 for some combinations can also be observed in Figure 6. The cementations which provided the highest percentage of no retrievability were also characterized by a higher value of total force, thus confirming that such luting agent–abutment couplings are not suitable for an easy retrieval.

## 5. Conclusions

The aim of this study was to determine how the luting agent and the abutment geometry influence the abutment–crown retrievability. For this purpose, realistic retrieve conditions were reproduced using Coronaflex^®^. The results show that the number of impulses which causes the failure of an abutment–coping cementation is affected by the aforementioned factors in a similar way to that which happens to the failure force in uniaxial tensile tests. However, the generated force during the impulsive stimulus did not reflect the dependence with the same steadiness. Moreover, the abutment geometry was found to be more crucial than the cement type in the stability of the coupling.

In general, combinations of short abutments and tapered shape resulted in cementation that was too weak, regardless of the luting agent. On the other hand, the most stable abutment geometry (i.e., 9 mm of height and cylindrical shape) provided non-retrievable copings with all cements. The authors suggest cementing crowns on a long abutment with a variable taper angle depending on the luting agent; temporary cements should be preferred with a more retentive shape, while a high taper angle should be used with permanent cements.

## Figures and Tables

**Table 1:** Number of impulses needed for retrieval: Kruskal–Wallis (KW) test results.

Factor	p
Cement	0.09
Height	2.22 × 10^−15^
Taper	5.17 × 10^−18^

**Table 2:** Average forces during retrieval: KW test results.

Factor	p
Cement	0.37
Height	1.62 × 10^−10^
Taper	0.28
